# Perceived inequalities and cultural inclusivity in UK Breast Cancer Information and Care: a mixed method study

**DOI:** 10.1016/j.jpra.2026.07.022

**Published:** 2026-07-27

**Authors:** Anna Chiara Corriero, Fay Fathima Imtiaz Fareed, Umar Rehman, Edelyne Tandanu, Agata J Baczynska, Paganiotis Antoniou, Freya Bakko, Ahmed Ezzat, Simon Filson, Manaf Khatib, Naveen Cavale, United Kingdom Plastics Research Collaborative Collaborators, Avani Desha Bhalla, Charlie Thompson, Charlotte Bairstow, Clara Calero Pages, Daniella Soussi, Danny Kazzazi, Deelan Vadher, Elena Brachimi, Eurekha Ravindran, Francis Banhidy, Frederick Wyatt, Jefferson George, Kailash Viswanathan, Karl Romain, Martina Schatz, Marvin Frommer, Meeral Makhdoom, Michael Grant, Natalia Makhdoom, Raveenjot Nagra, Reza Tahmid, Sai Sirikonda, Shazia Nusky, Sophie Mundell, Soyoung Lee, Stephanie Oh, Subham Roy, Vaishvi Dalal

**Affiliations:** a Department of Plastic Surgery, NHS Lothian, Edinburgh, UK; b Worcestershire Acute Hospitals NHS Trust, Redditch, UK; c Division of Surgery and Interventional Science, University College London, London, UK; d King’s College Hospital, London, UK; e Imperial College London, London, UK; f NHS Lincoln, Lincolnshire, UK; g Department of Plastic Surgery, Queen Victoria Hospital, East Grinstead, UK; h Department of Breast Surgery, Charing Cross Hospital, London, UK; i Department of Plastic Surgery, St Thomas’ Hospital, London, UK; j Department of Plastic Surgery, Lister Hospital, UK

**Keywords:** Breast cancer, Health inequities, Medical education, Patient safety

## Abstract

**Introduction:**

This study evaluates inequalities in National Health Service (NHS) breast cancer (BC) information, with a focus on equity and inclusivity. Drawing on the perspectives of UK medical students and resident doctors, the study explores perceived gaps in resources, supporting the development of inclusive materials that serve our diverse patient populations.

**Methods:**

A survey was distributed to gather insights into perceived gaps in education and resources. Following this, a comprehensive review of NHS BC leaflets was carried out, evaluating their inclusivity and representation. Based on these findings, an inclusive leaflet was designed; the leaflet’s readability was improved through a large language model.

**Results:**

A total of 1555 survey responses were collected; 60.3% (n=938) were medical students and 39.7% (n=617) were resident doctors. 85.5% (n=1330) of respondents denied attending breast-related cultural sensitivity training, and 64.7% (n=1006) felt inadequately equipped to address breast concerns in patients from diverse backgrounds.

The mean readability across NHS leaflets showed a readability age of 14-15 years. 15.9% (n=38) of resources being available in languages other than English (p=0.0012). While 27.6% (n=66) of the 239 NHS trusts had BC leaflets available, none was fully inclusive

These insights directly informed the design of a new leaflet to address identified gaps, focusing on inclusivity and readability. The clinician developed, LLM assisted leaflet met ages 12-13, closer to national recommended levels of 9-11. (FRES +7.72; FKGL -1.96; GFI -2.24; SMOG -1.4; CLI -0.39).

**Conclusions:**

Inequalities and lack of representation are seen at a national level within BC NHS patient materials. These inequalities are similarly perceived by students and resident doctors. This can have a negative impact on patient experience and journey through a BC diagnosis. Further work is necessary to improve the quality of information given to BC patients, with gaps bridged through inclusive resources and adequate preparation in BC counselling. For this purpose, the leaflet proposed in this paper represents a prototype that may inform the design of future studies and the development of more inclusive patient information resources, in line with NHS priorities.

## Introduction

Breast cancer (BC) makes up 15% of new cancers, the most common cancer in the United Kingdom, with over 50,000 cases annually.^1^ In females, it is predicted to have the second highest cancer mortality rate in 2025 (13.2 per 100,000).^2^ Early detection through self-examination, clinical exams and mammography is key^3^. The European Reference Organisation for Quality Assured Breast Screening (EUREF) states screening information must be accessible, relevant and tailored to the individual.^3^ Treatment and mortality risk information is population-specific and should be adapted for high-risk individuals with personal or family risk factors.

While communication inequalities exist across healthcare, BC is a useful model due to its prevalence, screening programme, and patient pathway, where clear information is essential from first contact. Management also has a strong surgical component: oncoplastic breast surgery combines tumour removal with reconstruction, placing the breast at the centre of oncological and aesthetic outcomes. Clear information is therefore vital to support decisions about reconstruction and care across public health, general practice, and breast and plastic surgery.

Previous UK literature highlights inequalities in breast care. Screening uptake varies by ethnicity: 67% of White British women attend first call, compared with 61% Indian, 43% Bangladeshi and 49% Black African women.^4^ Behavioural risk factors are also higher in LGBTQ+ groups (alcohol use, inactivity) and in people with disabilities (inactivity, obesity).^5^ Yet only those registered as female with their GP are invited to screening,^6^ and limited accessible equipment and locations delay diagnosis, often at advanced stages.^6^

Inequalities may also affect how BC information is communicated. Counselling must be individualised, accounting for cultural background and educational level to support understanding and informed decisions.^3^ A European study found 57.8% of women with HR+/HER2 BC were unaware of genomic testing.^7^, highlighting poor dissemination and understanding

Public health education shows similar gaps. In some countries, breast self-examination is underemphasised.^8^ UK and European data show women in deprived areas have lower incidence but higher mortality, likely due to comorbidities, reduced screening access, poorer education and suboptimal treatment.^9^ Screening uptake declines with increasing deprivation.^9^^,^^10^ Targeted measures, including accessible education and mobile mammography, are key to reducing mortality in these groups.^11^

Although information may be available online, patients may struggle to understand and follow it if not accessible.^12^ The UK’s average reading age is 9–11 years,^13^^,^^14^ meaning 43% of people struggle with health information.^12^ Healthcare professionals must therefore support patients from screening to diagnosis and counselling with clear explanations, supported by take-home materials that are understandable regardless of educational background. They should also signpost to reliable NHS or national platforms. Prevention and early detection, through counselling and breast self-examination education, are key to improving autonomy and early diagnosis.^3^

This study evaluates inequalities in NHS breast cancer (BC) information and care resources, focusing on access and inclusivity. Using perspectives from UK medical students and resident doctors, it explores gaps in resources, assesses inclusivity and readability, and supports development of accessible materials for diverse populations. The leaflet produced is a prototype, not for immediate clinical use.

## Methods

### Study design, setting and objectives

#### Study design and rationale behind the study

The study used a sequential mixed-methods design with three components: a survey of resident doctors/medical students, leaflet analysis, and development of a revised leaflet. Leaflet analysis provided an objective assessment of readability and inclusivity in existing NHS breast cancer materials, while the survey gave practice-informed insight into how these materials are perceived and where barriers to understanding and accessibility arise in clinical settings. Findings from both stages guided development of the new leaflet, addressing identified deficiencies; recurring survey concerns informed language simplification, structure, readability, and inclusivity in the prototype. This approach grounded the intervention in both measured shortcomings and real-world clinical experience.

Overall, the study follows a clear iterative logic: understanding the problem in practice (survey), diagnosing it (leaflet analysis), and developing a solution informed by both (leaflet development). This reflects a systems-to-practice-to-intervention pipeline, ensuring the final output is grounded in empirical assessment and real-world context.

A summary of this study rationale can be found below (Picture 1).

**Picture 1:**
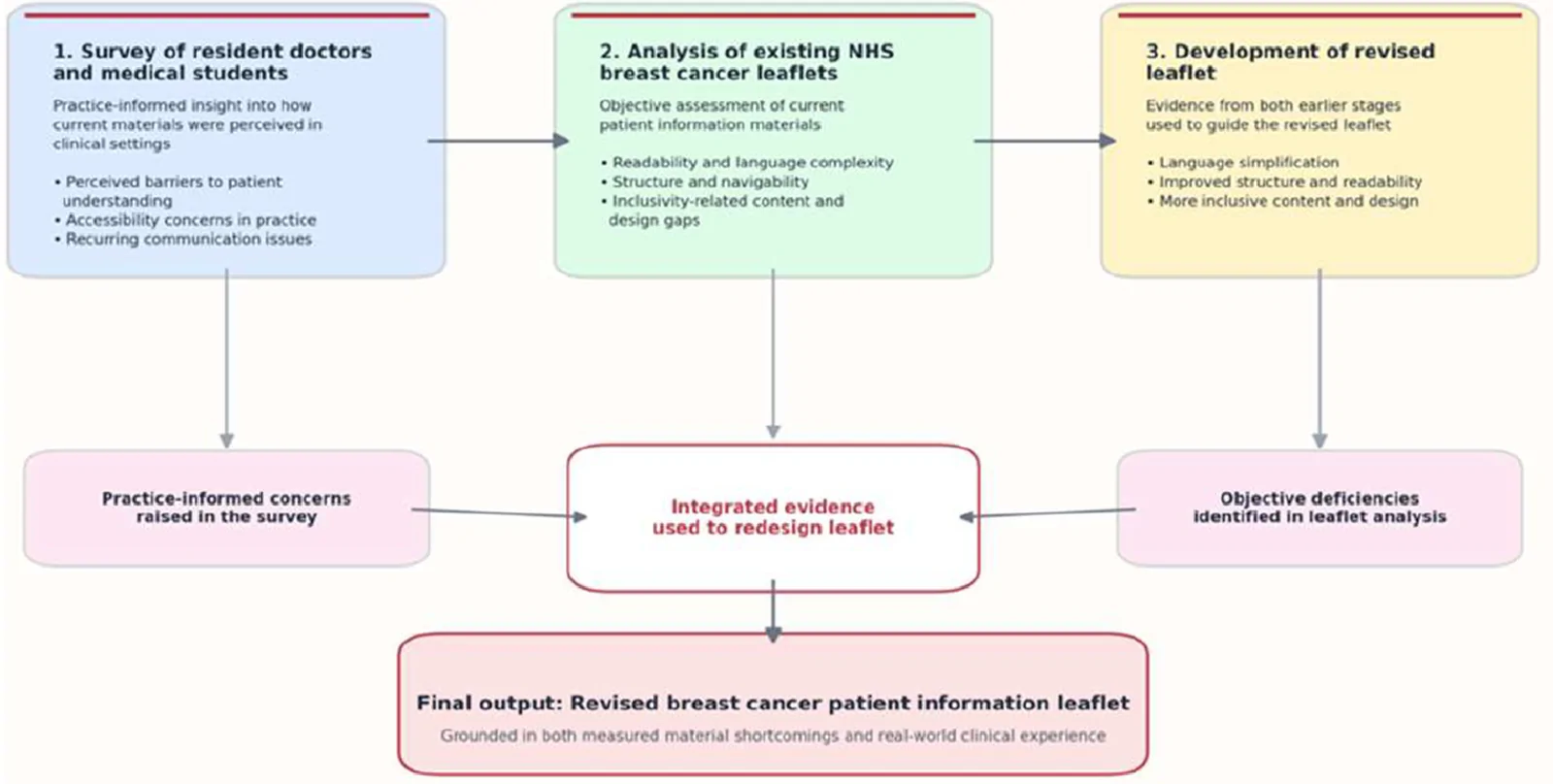
Flowchart summarising article rationale.

#### Survey study

To contextualise the need for inclusive, accessible breast cancer resources, a survey captured medical student and resident doctor perspectives on BC care, communication, and inclusivity of patient materials. The questionnaire was developed through literature review and a Delphi process involving students, residents and consultants (Appendix 1). It explored experiences of communication skills and exposure to inequalities in BC training. Data were collected June–August 2025 using a collaborator-based approach via the UK Plastics Research Collaborative (UKPRC), consistent with prior studies.^15^, ^16^, ^17^, ^18^

The primary objective was to explore experiences of BC counselling and identify gaps, including diversity and cultural sensitivity training. It also assessed availability and effectiveness of undergraduate teaching in building knowledge and confidence, and the accessibility of BC resources. These aims aligned with the overall goal of developing an equitable, patient-centred resource.

The study also aligns with several Top 10 priorities from the James Lind Alliance,^19^ including improving patient involvement in BC care and identifying needed information and support across the care pathway.

#### Survey study dissemination

The survey was disseminated via local leads in Foundation Schools, deaneries, and medical schools through social media and student society pages. Participants provided informed consent after reading the information sheet; without consent, no data were collected or analysed.

#### Survey study participants

This national cross-sectional survey followed STROBE guidelines.^20^ Eligible participants were UK medical students (≥18) and postgraduate doctors in training across all eighteen Foundation Schools during the 2024–2025 academic year. Doctors beyond registrar level, those withdrawn from training, or training outside the UK were excluded. A priori sample size calculations (using Cohen’s coefficients) aimed for >90% power, requiring at least 600 medical student and 600 resident doctor responses.

#### Survey variables

Predictor variables included demographic factors (gender, ethnicity and age); stage of training, gender and attitudinal factors (prior breast health training, clinic and theatre exposure, extra-curricular activities in breast surgery). Outcome variables included perceived inclusivity, readability and comprehensiveness of BC resources.

#### NHS leaflet analysis

The study analysed 312 BC-related leaflets from 239 UK-based healthcare trusts. Current leaflets were evaluated against predefined inclusivity criteria, with features such as demographic representation, language accessibility, and use of non-stereotypical language recorded and analysed descriptively; additionally, the leaflet analysis aimed to evaluate how many leaflets had accidentally stereotypical language.

A separate readability analysis of the NHS leaflets was also conducted. Readability scores were calculated for 312 NHS BC leaflets using established metrics such as Flesch Reading Ease (FRES), Flesch-Kincaid Grade Level (FKGL), Gunning Fog Index (GFI), SMOG index, and Coleman-Liau Index (CLI) through use of an online WebFX Readability tool (Pennsylvania, United States).^21^ Elements including headings, lists, and formatting artefacts were excluded to avoid false lowering of readability scores. Further, for comparison, a readability analysis of the clinician developed LLM assisted leaflet was carried out.

#### Design of the leaflet and readability improvement

To address gaps in accessibility and inclusivity, this study combined readability analysis of NHS leaflets with survey findings from medical students and resident doctors to inform development of a new resource. The leaflet was developed using existing literature and expert review from consultant breast surgeons via a Delphi consensus process. Designed as a prototype, it explores improvements in inclusivity and readability, with potential for future development and multilingual adaptation. As a consensus-based qualitative process, inter-rater reliability was not applied. The initial leaflet was created in Canva (Sydney, Australia)^22^ and uses green (Appendix 2) to avoid gendered colour associations. Key survey findings (e.g. limited multilingual resources, poor cultural representation, low readability confidence) directly informed design priorities, including simplified language, inclusive imagery, and adaptability for translation.

In line with NHS guidance that materials be understandable to a reading age of 9–11 years,^23^ a published prompt^24^^,^^25^ was applied using ChatGPT 5.1 (San Francisco, California, United States): “revise the provided patient education material to have a readability level comprehensible by an individual with the literacy level of an 11-year-old, while maintaining a tone suitable for an adult audience”. These align with standard thresholds (FRES ≥80; FKGL ≤6.9; GFI ≤6.9).^24^^,^^25^ The aim was to assess whether a simple, reproducible large language model intervention could improve readability towards NHS-recommended levels.

#### Statistical analysis

Statistical analyses were conducted using Stata version 19 (College Station, United States).^26^ A mixed-methods analytical approach combining quantitative survey analysis with objective content evaluation and readability metrics was carried out. Descriptive statistics were reported as frequencies and percentages for categorical variables and as means with standard deviation for ordinal variables. Group comparisons were assessed using the Wilcoxon rank-sum test as non-parametric data was generated. Significance was set at p<0.05.

## Results

### Survey demographics

The survey included 1555 participants: 60.3% medical students (n=938) and 39.7% resident doctors (n=617). 56.5% were female (n=879) and 42.8% male (n=665). Ethnic groups were diverse, most commonly White British (24.1%), British Indian (19.6%), Other White (13.5%), British Pakistani (7.9%), and other Asian backgrounds (7.3%) (Table 1).

**Table 1:** Demographics of respondents.

Category	Subcategory	Count	Percentage
Total answers	Total	1555	100%
Medical students	Year 1	73	4.7%
	Year 2	120	7.8%
	Year 3	207	13.3%
	Intercalating	47	3.0%
	Penultimate year	294	18.9%
	Final year	200	12.9%
	Total	938	60.3%
Resident Doctors	Foundation year 1	144	9.3%
	Foundation year 2	142	9.1%
	Core Surgical trainee year 1	47	3.0%
	Core surgical trainee year 2	67	4.3%
	Clinical fellows, locally employed doctors, trust grade senior house officers	221	14.2%
	Total	617	39.7%
Gender	Female	879	56.5%
	Male	665	42.8%
	Non-Binary	7	0.5%
	Prefer not to say	13	0.8%
Age	18-24	836	53.8%
	25-30	607	39.0%
	30+	112	7.2%

Undergraduate exposure was mainly through compulsory lectures (74.9%, n=1164), with fewer experiencing placements (49.5%, n=770) or theatre sessions (42.5%, n=661); similar gaps existed in postgraduate training (Appendix 3). Confidence in breast-related clinical skills varied (Appendix 4), with higher confidence in examinations among female respondents than males (4.51 vs 4.32, p=0.001), and variation across ethnicities.

Training in communication was limited (Table 2). While 79.7% (n=1240) had training in delivering a cancer diagnosis, only 42.6% (n=663) had BC-specific counselling experience and 14.5% (n=225) received cultural sensitivity training. This contrasts with GMC guidance emphasising difficult and inclusive communication.^27^

**Table 2:** Communication training levels amongst participants*. P values were calculated using chi-square tests comparing medical students and resident doctors. Three responses with conflicting participant-category data were excluded from subgroup analysis.

Category	Medical students all, Yes	Medical students all, No	Resident doctors all, Yes	Resident doctors all, No	P value
Have you been trained in breaking bad news to patients when it comes to cancer diagnosis in medical school?	685 (73.2%)	251 (26.8%)	552 (89.6%)	64 (10.4%)	<0.001*
Have you had opportunities to counsel patients on breast health during your clinical rotations?	341 (36.4%)	595 (63.6%)	319 (51.8%)	297 (48.2%)	<0.001*
Do you feel prepared to address breast health concerns in patients from diverse cultural backgrounds?	247 (26.4%)	689 (73.6%)	301 (48.9%)	315 (51.1%)	<0.001*
Have you received training on cultural sensitivity related to discussing breast health with patients?	107 (11.4%)	829 (88.6%)	116 (18.8%)	500 (81.2%)	<0.001*

Experiences with BC patient information showed gaps (Table 3): only 18.9% (n=297) encountered multilingual leaflets and 24.7% (n=387) culturally inclusive materials. Perceived equity in BC care was low (Table 4), with mean scores ≤3, indicating neutral to negative views on inclusivity.

**Table 3:** Inclusivity of breast resources. P values were calculated using chi-square tests comparing medical students and resident doctors. Three responses with conflicting participant-category data were excluded from subgroup analysis.

Category	Response	Medical students n (%)	Resident doctors n (%)
I have seen BC information leaflets in clinical or educational settings	Yes	641 (68.5%)	418 (67.9%)
	No	198 (21.2%)	92 (14.9%)
	I am not sure / I have not seen any BC leaflets on placement	97 (10.4%)	106 (17.2%)
The BC leaflets I have seen are inclusive of people from different ethnic and cultural backgrounds	Yes	231 (24.7%)	150 (24.4%)
	No	202 (21.6%)	92 (14.9%)
	I am not sure / I have not seen any BC leaflets on placement	503 (53.7%)	374 (60.7%)
The BC leaflets I have seen are available in multiple languages	Yes	155 (16.6%)	138 (22.4%)
	No	291 (31.1%)	133 (21.6%)
	I am not sure / I have not seen any BC leaflets on placement	490 (52.4%)	345 (56.0%)
The leaflets are appropriate for the average UK reading age (9 to 11 years old)	Yes	383 (40.9%)	262 (42.5%)
	No	125 (13.4%)	39 (6.3%)
	I am not sure / I have not seen any BC leaflets on placement	428 (45.7%)	315 (51.1%)
The leaflets are widely accessible in clinical settings	Yes	439 (46.9%)	271 (44.0%)
	No	100 (10.7%)	60 (9.7%)
	I am not sure / I have not seen any BC leaflets on placement	397 (42.4%)	285 (46.3%)
I feel confident that patients from all backgrounds can understand and engage with the BC materials provided	Yes	163 (17.4%)	119 (19.3%)
	No	263 (28.1%)	150 (24.4%)
	I am not sure / I have not seen any BC leaflets on placement	510 (54.5%)	347 (56.3%)

**Table 4:** Assessment of Perceived Equity in Breast Care Delivery. Scale: 0 = I have not observed breast care services (in primary or secondary care) hence I am unable to respond. 1 = Strongly disagree, 2 = Disagree, 3 = Neutral, 4= Agree and 5 = Strongly agree.

Question	Category	Result	Category	Result	Category	Result	Category	Result
Breast care services in my setting are inclusive of patients from all ethnic backgrounds.	Females (mean, SD)	2.48 (1.82)	Pre-clinical student (mean, SD)	1.61 (1.83)	Medical student (mean, SD)	2.34 (1.76)	White British (mean, SD)	2.48 (1.87)
	Males (mean, SD)	2.49 (1.75)	Clinical student (mean, SD)	2.99 (1.41)	Resident doctor (mean, SD)	2.69 (1.81)	British Indian (mean, SD)	2.35 (1.82)
	P value	0.8689	P value	<0.001*	P value	<0.001*	P value	0.1587
Breast care is delivered equitably regardless of patients' ethnicity.	Females (mean, SD)	2.56 (1.87)	Pre-clinical student (mean, SD)	1.68 (1.91)	Medical student (mean, SD)	2.42 (1.84)	White British (mean, SD)	2.51 (1.88)
	Males (mean, SD)	2.54 (1.80)	Clinical student (mean, SD)	3.09 (1.48)	Resident doctor (mean, SD)	2.73 (1.82)	British Indian (mean, SD)	2.45 (1.85)
	P value	0.6832	P value	<0.001*	P value	0.008*	P value	0.1587
I believe breast care pathways are sensitive to the needs of underrepresented groups (e.g., ethnic minorities, migrants, lower socioeconomic status).	Females (mean, SD)	2.10 (1.60)	Pre-clinical student (mean, SD)	1.35 (1.56)	Medical student (mean, SD)	2.02 (1.54)	White British (mean, SD)	2.11 (1.63)
	Males (mean, SD)	2.24 (1.60)	Clinical student (mean, SD)	2.63 (1.25)	Resident doctor (mean, SD)	2.37 (1.67)	British Indian (mean, SD)	2.03 (1.63)
	P value	0.0661	P value	<0.001*	P value	<0.001*	P value	0.0012*
My education or training has included discussions about inequalities in breast cancer care.	Females (mean, SD)	1.62 (1.47)	Pre-clinical student (mean, SD)	1.15 (1.42)	Medical student (mean, SD)	1.48 (1.34)	White British (mean, SD)	1.55 (1.36)
	Males (mean, SD)	1.61 (1.37)	Clinical student (mean, SD)	1.78 (1.20)	Resident doctor (mean, SD)	1.82 (1.52)	British Indian (mean, SD)	1.73 (1.52)
	P value	0.7133	P value	<0.001*	P value	0.004*	P value	0.3854
I have seen differences in how breast care is offered or accessed by patients from underrepresented backgrounds.	Females (mean, SD)	1.50 (1.37)	Pre-clinical student (mean, SD)	1.21 (1.38)	Medical student (mean, SD)	1.38 (1.27)	White British (mean, SD)	1.44 (1.34)
	Males (mean, SD)	1.52 (1.38)	Clinical student (mean, SD)	1.62 (1.13)	Resident doctor (mean, SD)	1.69 (1.50)	British Indian (mean, SD)	1.61 (1.43)
	P value	0.7896	P value	<0.001*	P value	<0.001*	P value	0.0063*

Only 18.3% (n=287) felt current materials were understandable across backgrounds, and 41.4% (n=649) considered them appropriate for the UK reading age (Table 3).

### Leaflet analysis

A total of 239 NHS trust websites were reviewed (Appendix 5). Most (71.5%, n=173) had no leaflets; 18.0% (n=43) had some resources, 9.2% (n=22) videos, and 27.2% (n=65) links to external content (e.g. NHS or cancer charities). Overall, 66 trusts (27.6%) across the UK had patient leaflets, totalling 312, analysed per trust as a package.

Comparing perceptions with objective findings (Table 5) showed clear gaps. While 24.7% (n=387) of respondents reported diverse representation, objective analysis found this in 40.9% (n=27) of trusts with leaflets—only 11.3% of all trusts (Z=16.7, p<0.0001). Similarly, 18.9% (n=297) reported seeing non-English leaflets, versus 57.6% (n=38) of leaflet-providing trusts (15.9% overall) (Z=3.2, p=0.0012).

**Table 5:** Inclusivity of analysed breast resources. This shows an analysis of all trust resources for inclusivity, represented trust by trust; overall, this showed no NHS downloadable resource to be fully inclusive from all perspectives.

Question	Yes(Count, Percentage)	No(Count, Percentage)
Does the trust’s resource (group of leaflets) acknowledge that breast cancer can affect people of all genders, not just women?	7 (10.6%)	59 (89.4%)
Are gender-neutral terms used where appropriate (e.g., “people with breast cancer” vs. “women with breast cancer”)?	32 (48.5%)	34 (51.5%)
Are examples, images, or illustrations representative of diverse racial or ethnic backgrounds?	27 (40.9%)	39 (59.1%)
Is language culturally sensitive and free from stereotypes?	64 (97.0%)	2 (3.0%)
Is there a mention or availability of resources in a language other than English?	38 (57.6%)	28 (42.4%)
Do images depict scenarios inclusive of doctors/nurses/healthcare professionals from different backgrounds (ethnicity)?	18 (27.3%)	48 (72.3%)
Are there references or advice that could be offensive or exclusionary to people of different religions or cultural practices?	0 (0.0%)	66 (100.0%)
Are accommodations or options mentioned for religious/cultural practices that may affect treatment or support (e.g., dietary restrictions, modesty considerations)?	0 (0.0%)	66 (100.0%)
Do images depict scenarios inclusive of doctors/nurses/healthcare professionals from different religions ?	4 (6.1%)	62 (93.9%)
Is language inclusive of sexual orientation and family structures (e.g., “partner” instead of assuming “husband/wife”)?	11 (1.5%)	65 (98.5%)
LGBTQ+ patient mentions or accommodations	3 (4.5%)	63 (95.4%)
Do the images or illustrations in the resources represent a diverse range of body types (e.g., different shapes, sizes, and physical appearances)?	18 (27.3%)	48 (72.7%)
Are there any other elements in the leaflet that could exclude or marginalise specific groups? (free text)	3 (4.6%)	63 (95.4%)

### Readability analysis of current NHS leaflets

The NHS leaflets demonstrated a large and inconsistent variation in scores with FRES ranging between 15-88 corresponding to approximately 9- 18+ years of age. (Table 6). The mean readability scores across the NHS leaflets were: FRES 56.78 ± 11.5 (ages 14-16), FKGL 9.36 ± 1.91 (ages 14-15), GFI 12.24 ± 2.15 (ages 17-18), SMOG 8.80 ± 1.59 (ages 13-15), and CLI 13.09 ± 2.38 (ages 18-19). NHS recommendations of health literacy toolkits are to aim for a reading age of 9 to 11 years old.^13^^,^^23^

**Table 6:** Readability assessment of existent NHS leaflets. Legend: For FRES: higher = easier to read. For FKGL, GFI, SMOG, CLI: lower = lower reading grade level.

Measure	NHS leaflets mean (n = 312)	Our leaflet (clinician + LLM assisted)	Difference
FRES Flesch Reading Ease	56.78	64.5	7.72 ↑
FKGL Flesch-Kincaid Grade Level	9.36	7.4	−1.96 ↓
GFI Gunning Fog Index	12.24	10	−2.24 ↓
SMOG Simple Measure of Gobbledygook	8.8	7.4	−1.40 ↓
CLI Coleman-Liau Index	13.09	12.7	−0.39 ↓

### Readability analysis of newly developed prototype leaflet

In comparison, our clinician-developed leaflet whose readability was improved through a Large Language Model (LLM) reflected scores of: FRES 64.5, FKGL 7.4, GFI 10.0, SMOG 7.4, and CLI 12.7. This indicated an increase in reading ease and reduction in required reading levels of approximately two years in comparison with the mean reading age of NHS materials. Differences in score improvement are also highlighted within tablet 6.

Our LLM assisted leaflet met the readability level of ages 12-13, significantly closer to national recommended levels; compared to NHS leaflets (average age 14-15). As only a single clinician-developed leaflet was available, formal statistical comparison was limited.

## Discussion and conclusion

### Discussion

#### Principal findings

This study highlights limited inclusivity in UK breast cancer patient resources, consistent with survey findings showing low confidence in caring for minority groups. Many respondents had not encountered culturally or linguistically appropriate materials, aligning with the leaflet evaluation.

Readability analysis showed most NHS BC leaflets exceed recommended reading levels, limiting access for many patients. Use of LLMs to improve readability should be explored, as they can significantly simplify language for target populations. Importantly, the leaflet was clinician-led to ensure accuracy, with LLMs used only to improve clarity and readability while maintaining content. Breadth was balanced with readability to better match the UK average reading age.

The inclusive, more readable leaflet addresses these gaps as a prototype for future development. Alignment between survey findings and leaflet analysis strengthens its evidence base, targeting real-world gaps identified by healthcare professionals.

#### Comparison with existing literature and interpretation

Our analysis of 239 NHS Trust websites (Appendix 5) found no fully inclusive downloadable BC patient materials. Only 27.6% of trusts had online leaflets; just 15.9% offered multilingual versions and 11.3% included diverse racial or ethnic representation, consistent with survey findings. Readability was also a concern, with only 41.4% of respondents agreeing materials matched public reading levels. Earlier UK studies similarly found BC materials unsuitable for average reading age,^28^ with later work showing only 63% met readability standards,^12^ suggesting persistent exclusion of lower-literacy groups.

These findings reinforce the need for more inclusive, readable BC resources, such as the prototype leaflet developed here, with further gains possible using large language models (Appendix 2). Survey results also align with evidence of clinician uncertainty when treating minority groups,^29^ reflecting broader healthcare inequalities seen in conditions like chronic respiratory disease^30^ and dementia care.^31^

This is concerning given UK demographics, with over a quarter of the population not identifying as White British,^32^ and misalignment with NHS priorities. The NHS Patient and Carer Race Equality Framework^33^ commits to inclusive care through cultural awareness and co-creation of resources.

Gaps in patient materials may reduce engagement and contribute to higher rates of advanced BC in ethnic minority women, including Black African (25%) and Black Caribbean women (22%) versus White British women (13%).^34^ Early detection is key to improving outcomes,^35^ yet barriers persist, including low symptom awareness, language and literacy challenges, misconceptions about screening, and lack of confidence with healthcare professionals.^36^^,^^37^^,^^38^ Cultural and religious factors, such as stigma, modesty, and preference for female clinicians, further delay help-seeking,^36^^,^^37^^,^^39^^,^^40^ contributing to lower screening uptake and poorer outcomes in these groups.^4^^,^^34^^,^^36^

#### Generalisability

The NHS Long Term Plan^41^ commits over £1 billion to reduce inequalities and modernise screening, aiming for 75% of cancers to be diagnosed at stages I–II by 2028. However, this relies on equitable access to culturally sensitive care and information to improve engagement and outcomes.^42^^,^^43^ Despite major investment in treatment technologies, less focus has been placed on communication.^44^ While inequalities are systemic, improving patient resources is a practical starting point, particularly in breast cancer where disparities are well documented.

Redeveloping resources to be linguistically and culturally sensitive is key to improving understanding and screening uptake.^44^ A meta-analysis^45^ showed such interventions increased mammography and pap smear uptake by 18% and 54%, highlighting their impact on engagement and outcomes.

These improvements are achievable across NHS resources, not just BC. Future work could use large language models to enhance readability, translation, and accessibility (e.g. audio), widening reach. Alongside this, better oncological and cultural competence training is needed to equip clinicians to communicate effectively with diverse patients, especially as early BC presentations often occur outside specialist settings.

#### Recommendations and future directions

Future work developing patient information leaflets for clinical deployment should incorporate co-development with patient and public involvement^40^, ^46^ groups alongside clinical experts, with LLMs used as supportive tools for refinement rather than primary content generation. In addition, formal comparative studies assessing performance against existing NHS breast cancer patient information leaflets in real-world clinical settings are required to establish external validity and patient-centred effectiveness. However, these evaluations were beyond the scope of the present proof-of-concept study.

Additionally, the current state of oncological training and service provision in the UK requires a comprehensive approach to address its shortcomings and achieve commitments set out on the NHS Long Term Plan.^41^ Furthermore, greater emphasis should be placed on the cultural and linguistic diversity demonstrated in health campaigns and patient resources, which have been shown to be exclusionary and not representative (Table 5).

#### Strengths and limitations

This study has several strengths, including achieving a priori sample size targets and aligning with NHS priorities and patient needs. However, limitations exist. Only online leaflets were analysed, excluding paper resources used in clinical settings. Despite this, it is the first study to comprehensively assess readability and inclusivity of NHS BC resources. Future work should include patient participation groups and multicentre surveys across diverse populations. Studies assessing patient-reported readability, as well as evaluating the effectiveness of new versus existing resources before wider implementation, would be valuable.

Survey limitations include reliance on self-reported data, with potential recall bias. Convenience and snowball sampling may have introduced bias, and survey flow metrics (e.g. response rate, duplicates, incomplete responses) could not be calculated. The cross-sectional design reflects a snapshot in time, and variation in responses likely relates to differing clinical exposure, with many participants not directly involved in BC care.

AI use is both a strength and limitation. While enabling innovation, it lacks clinical nuance and may reflect biases in training data, risking underrepresentation of diverse groups. Improvements in readability scores do not necessarily translate to better understanding or engagement, and do not account for variation in literacy or patient preferences, which require further evaluation.

## Conclusions

In conclusion, this study has demonstrated lack of inclusion in the patient-oriented BC resources. In line with the NHS long-term plan and priorities, it is paramount that these undergo frequent review and improvement, to reflect the cultural and linguistic make-up of the population; this will ensure delivery of accurate, relatable and understandable information to all patient groups. The developed leaflet presented in this work was created to achieve the above discussed goals of patient involvement and education in breast cancer decisions. Implementing these measures will not only enhance patient engagement and early detection, but also contribute to narrowing persistent disparities in BC outcomes. Alongside this, continuous improved education and cultural competence development must be provided at different stages of training, to positively contribute to BC patient outcomes.

## Declaration of generative AI and AI-assisted technologies in the manuscript preparation process

During the preparation of this work the author(s) used ChatGPT in order to improve the readability of the newly developed patient information material. This has been previously published in the literature (22,23). After using this tool/service, the authors reviewed and edited the content as needed and take full responsibility for the content of the published article.

## Funding

None.

## Ethical approval

The study received ethical approval from the Research Ethics Office at Kings College London on 03/06/2025, with the ethical approval reference being MRSU-24/25-49546. This study is compliant with the General Data Protection Regulation (2016/679) and the Data Protection Act (2018) requirements. All investigators and study site members will comply with the requirements of the General Data Protection Regulation (2016/679). No personal or identifiable information was collected during the survey study component and all data was anonymised.

## Declaration of competing interest

The authors declare no conflict of interest.

## Appendix Supplementary materials

Image, application 1
