# Supplementary material for: Perceived inequalities and cultural inclusivity in UK Breast Cancer Information and Care: a mixed method study
Source: JPRAS Open. 2026 Jul 27;51:710–22. doi: 10.1016/j.jpra.2026.07.022 (PMC13508653; doi:10.1016/j.jpra.2026.07.022)
Supplement: Supplementary file 1 [file mmc1.docx]

Appendices

**Appendix 1: Study Survey Codebook**

### **Section 1: Consent and Eligibility**

| **Variable Name** | **Question Text** | **Type** | **Response Options** | **Coding / Notes** |
| --- | --- | --- | --- | --- |
| consent_use | I consent for my answer to be used for research purposes including presentations and publications by the United Kingdom Plastics Research Collaborative. | Multiple Choice | Yes, No | 1=Yes, 0=No |
| uk_residency | I confirm that I am based in the United Kingdom as a student or a resident doctor. | Multiple Choice | I confirm, I am not based in the UK | 1=Confirm, 0=Not UK-based |

### **Section 2: Demographics**

| **Variable Name** | **Question Text** | **Type** | **Response Options** | **Coding / Notes** |
| --- | --- | --- | --- | --- |
| referrer | What is the name of the collaborator who referred you to the study? | Text | Open-ended | Leave blank if none |
| role | Which of these represents you best? | Multiple Choice | Medical student, Resident Doctor | 1=Med Student, 2=Resident Doctor |
| med_year | If you are a medical student, what year are you in? | Multiple Choice | Year 1, Year 2, Year 3, Year 4, Year 5, Intercalating year, I am a resident doctor | 1=Year1, 2=Year2, … 6=Intercalating, 7=Resident |
| doc_type | If you are a resident doctor, which describe you best? | Multiple Choice | FY1, FY2, CST1, CST2, Clinical Fellow/Trust Grade, I am a medical student | 1=FY1, 2=FY2, 3=CST1, 4=CST2, 5=Fellow/Trust, 6=Med Student |
| gender | What is your gender? | Multiple Choice | Male, Female, Prefer not to say, Non-binary | 1=Male, 2=Female, 3=Prefer not to say, 4=Non-binary |
| age_group | Please select your age group | Multiple Choice | 18-24, 25-30, 30+ | 1=18-24, 2=25-30, 3=30+ |
| sec_school | Secondary school attended | Multiple Choice | State, Comprehensive, Grammar, Private | 1=State, 2=Comprehensive, 3=Grammar, 4=Private |
| ethnicity | What is your ethnicity? | Multiple Choice | [All provided options] | Code each as 1…N, include “Prefer not to say” |
| med_school | Current or graduated medical school | Multiple Choice | [All provided options, UK & non-UK] | Assign numeric codes sequentially |
| deanery | If a junior doctor, what deanery do you work in? | Multiple Choice | [All provided options] | Assign numeric codes; Med Student = 0 |

### **Section 3: Confidence in Breast Surgery Skills**

**Likert Scale for most questions:** 1 = Not at all confident … 7 = Extremely confident

| **Variable Name** | **Question Text** | **Type** | **Response Options** | **Coding / Notes** |
| --- | --- | --- | --- | --- |
| conf_exam | How confident are you in performing a breast exam? | Likert | 1–7 | 1=Not at all, 7=Extremely |
| conf_history | How confident are you in taking a breast history? | Likert | 1–7 | Same coding |
| conf_diag | How confident are you in explaining a breast cancer diagnosis? | Likert | 1–7 | Same coding |
| conf_patient_ed | How comfortable are you educating patients on self-exam? | Likert | 1–7 | Same coding |
| conf_triple | How confident explaining triple assessment? | Likert | 1–7 | Same coding |
| conf_screening | How confident explaining UK breast screening? | Likert | 1–7 | Same coding |
| conf_redflags | How confident identifying red flags? | Likert | 1–7 | Same coding |
| conf_2ww | How confident identifying 2-week wait patients? | Likert | 1–7 | Same coding |
| conf_family | How confident in counselling for family screening? | Likert | 1–7 | Same coding |
| conf_anatomy | How confident in breast anatomy, vasculature, lymphatics? | Likert | 1–7 | Same coding |
| conf_sensitive | How confident discussing sensitive topics (e.g., diagnosis)? | Likert | 1–7 | Same coding |
| conf_emotion | Comfort addressing patients’ emotional responses? | Likert | 1–7 | Same coding |
| conf_roles | Familiarity with roles of healthcare professionals? | Likert | 1–7 | 1=Not at all … 7=Extremely |
| trained_badnews | Trained in breaking bad news in medical school? | Multiple Choice | Yes, No | 1=Yes, 0=No |
| counsel_clinical | Had opportunities to counsel patients on breast health? | Multiple Choice | Yes, No | 1=Yes, 0=No |
| prepared_diverse | Feel prepared to address diverse cultural backgrounds? | Multiple Choice | Yes, No | 1=Yes, 0=No |
| training_cultural | Received cultural sensitivity training? | Multiple Choice | Yes, No | 1=Yes, 0=No |
| mdt_undergrad | Participated in MDT meetings during undergrad? | Multiple Choice | Yes, No | 1=Yes, 0=No |
| resources_needed | Resources that could increase confidence | Checkbox | Short videos, Leaflets, Written guides, More placements, Voluntary placements, Social media, Other | Multiple binary-coded variables |

### **Section 4: Inclusivity in Breast Care**

**Likert Scale:** 0 = Have not observed … 5 = Strongly agree

| **Variable Name** | **Question Text** | **Type** | **Response Options** | **Coding / Notes** |
| --- | --- | --- | --- | --- |
| incl_ethnic | Breast care services inclusive of all ethnic backgrounds | Likert | 0–5 | 0=Not observed, 1–5 = disagree–agree |
| incl_equity | Breast care delivered equitably | Likert | 0–5 | Same coding |
| incl_sensitive | Pathways sensitive to underrepresented groups | Likert | 0–5 | Same coding |
| incl_diff_access | Observed differences in access by underrepresented groups | Likert | 0–5 | Same coding |
| incl_training | Education included inequalities discussion | Likert | 0–5 | Same coding |

### **Section 5: Breast Cancer Information Materials**

| **Variable Name** | **Question Text** | **Type** | **Response Options** | **Coding / Notes** |
| --- | --- | --- | --- | --- |
| seen_leaflets | Seen breast cancer information leaflets | Multiple Choice | Yes, No, I am not sure | 1=Yes, 0=No, 9=Not sure |
| inclusive_leaflets | Leaflets inclusive of diverse ethnic/cultural backgrounds | Multiple Choice | Yes, No, I am not sure | 1=Yes, 0=No, 9=Not sure |
| leaflets_languages | Leaflets available in multiple languages | Multiple Choice | Yes, No, I am not sure | 1=Yes, 0=No, 9=Not sure |
| leaflets_resources_seen | Resources seen | Checkbox | NHS leaflets, Macmillan, GP/hospital info, Online, Social media, None, Other | Multiple binary-coded variables |
| leaflets_reading_age | Appropriate reading age | Multiple Choice | Yes, No, I am not sure | 1=Yes, 0=No, 9=Not sure |
| leaflets_relatable | Materials reflect diverse patients | Multiple Choice | Yes, No, I am not sure | 1=Yes, 0=No, 9=Not sure |
| leaflets_accessible | Widely accessible | Multiple Choice | Yes, No, I am not sure | 1=Yes, 0=No, 9=Not sure |
| confident_patient_engage | Confident patients can engage with materials | Multiple Choice | Yes, No, I am not sure | 1=Yes, 0=No, 9=Not sure |

### **Section 6: Exposure to Breast Surgery**

| **Variable Name** | **Question Text** | **Type** | **Response Options** | **Coding / Notes** |
| --- | --- | --- | --- | --- |
| activities_medschool | Which activities completed in med school? | Checkbox | Compulsory clinics, Theatre, SSC, Society events, Official events, Intercalation, Lectures, Other | Multiple binary-coded |
| activities_confidence | Did activities increase confidence? | Multiple Choice | Yes, No, Not undertaken | 1=Yes, 0=No, 9=Not undertaken |
| compulsory_placement | Had compulsory breast surgery placement? | Multiple Choice | Yes, No | 1=Yes, 0=No |
| confirm_role | Confirm student or doctor | Multiple Choice | Med student, Resident doctor | 1=Med Student, 2=Resident Doctor |

### **Section 7: Postgraduate Doctors Only**

| **Variable Name** | **Question Text** | **Type** | **Response Options** | **Coding / Notes** |
| --- | --- | --- | --- | --- |
| trained_badnews_postgrad | Trained in breaking bad news post-FY? | Multiple Choice | Yes, No | 1=Yes, 0=No |
| teaching_fy | Received compulsory teaching FY1+ | Multiple Choice | Yes, No | 1=Yes, 0=No |
| mdt_postgrad | Participated in MDT FY1+ | Multiple Choice | Yes, No | 1=Yes, 0=No |
| rotation_fy | Rotation in breast surgery FY1+ | Multiple Choice | Yes, No | 1=Yes, 0=No |
| cover_outhours | Cover breast surgery out of hours FY1+ | Multiple Choice | Yes, No | 1=Yes, 0=No |
| rotation_cst | CST rotation in breast surgery | Multiple Choice | Yes, No, Not CST | 1=Yes, 0=No, 9=Not CST |

Appendix 2

**Appendix 3: Exposure to undergraduate and postgraduate activities**

| **Activity** | **Yes (count, percentage)** | **No (count, percentage)** | **Total** |
| --- | --- | --- | --- |
| **Undergraduate activities** |  |  |  |
| Compulsory lectures about breast conditions | 1164 (74.9%) | 391  (25.2%) | 1555 |
| Compulsory breast surgery placement (clinics) | 770  (49.5%) | 785  (50.5%) | 1555 |
| Compulsory breast surgery placement (theatre sessions) | 661  (42.5%) | 894  (57.5%) | 1555 |
| University society events about breast surgery | 226  (14.5%) | 1329  (85.5%) | 1555 |
| student selected component in breast surgery | 119  (7.6%) | 1436  (92.3%) | 1555 |
| Official breast surgery events (for example by the Association of Breast Surgery) | 71  (4.6%) | 1484  (95.4%) | 1555 |
| Intercalation in breast surgery | 22  (1.4%) | 1533  (98.6%) | 1555 |
| **Postgraduate activities** |  |  |  |
| In your postgraduate training (after the beginning of foundation years), have you been trained in breaking bad news to patients when it comes to cancer diagnosis? | 323  (52.9% | 288  (47.1%) | 611 |
| Have you received any compulsory teaching in FY1 or beyond about breast conditions and surgery? | 193  (31.6%) | 417  (68.3%) | 610 |
| Have you participated in multidisciplinary team meetings discussing breast cancer cases in FY1 or beyond? | 141  (23.1%) | 469  (76.9%) | 610 |
| Have you had or will you have a rotation in breast surgery in FY1 or beyond? | 85  (13.9%) | 525  (86.1%) | 610 |
| Have you had or will you have to cover breast surgery out of hours (for example as part of a general surgery rotation) in FY1 or beyond? | 219  (35.9%) | 391  (64.1%) | 610 |
| If you are a core surgical trainee, have you had or will you have a rotation in breast surgery during CST? | 40 (24.1%) | 126  (75.9%) | 166 |

**Appendix 4:** Self-reported confidence with breast-cancer related clinical tasks

Scale: 1. Not at all confident

2. Slightly confident

3. Somewhat confident

4. Moderately confident

5. Fairly confident

6. Very confident

7. Extremely confident

| Question | Category | Score | Category | Score | Category | Score | Category | Score |
| --- | --- | --- | --- | --- | --- | --- | --- | --- |
| How confident are you in performing a Breast exam? | Females (mean, SD) | 4.51 (1.60) | Pre-clinical student (mean, SD) | 3.24 (1.69) | Medical student (mean, SD) | 4.06 (1.68) | White British (mean, SD) | 4.56 (1.51) |
|  | Males (mean, SD) | 4.32 (1.50) | Clinical student (mean, SD) | 4.82 (1.22) | Resident doctor (mean, SD) | 4.98 (1.17) | British Indian (mean, SD) | 4.28 (1.75) |
|  | P value | **0.001*** | P value | **<0.001*** | P value | **<0.001*** | P value | **0.01597*** |
| How confident are you in taking a Breast history? | Females (mean, SD) | 4.67 (1.57) | Pre-clinical student (mean, SD) | 3.60 (1.67) | Medical student (mean, SD) | 4.29 (1.68) | White British (mean, SD) | 4.80 (1.49) |
|  | Males (mean, SD) | 4.52 (1.55) | Clinical student (mean, SD) | 4.99 (1.32) | Resident doctor (mean, SD) | 5.08 (1.23) | British Indian (mean, SD) | 4.43 (1.78) |
|  | P value | **0.0167*** | P value | **<0.001*** | P value | **<0.001*** | P value | **0.0177*** |
| Confidence in explaining a breast cancer diagnosis | Females (mean, SD) | 3.91 (1.61) | Pre-clinical student (mean, SD) | 2.94 (1.44) | Medical student (mean, SD) | 3.64 (1.57) | White British (mean, SD) | 3.91 (1.62) |
|  | Males (mean, SD) | 4.00 (1.51) | Clinical student (mean, SD) | 4.27 (1.38) | Resident doctor (mean, SD) | 4.41 (1.45) | British Indian (mean, SD) | 3.84 (1.67) |
|  | P value | 0.4713 | P value | **<0.001*** | P value | **<0.001*** | P value | 0.1998 |
| How comfortable are you in educating patients about breast self-examinations and the importance of early detection? | Females (mean, SD) | 4.49 (1.61) | Pre-clinical student (mean, SD) | 3.41 (1.65) | Medical student (mean, SD) | 4.02 (1.62) | White British (mean, SD) | 4.37 (1.62) |
|  | Males (mean, SD) | 4.02 (1.58) | Clinical student (mean, SD) | 4.57 (1.38) | Resident doctor (mean, SD) | 4.69 (1.52) | British Indian (mean, SD) | 4.23 (1.72) |
|  | P value | **<0.001*** | P value | **<0.001*** | P value | **<0.001*** | P value | **0.00247*** |
| How confident are you in explaining to a patient how triple assessments through secondary care referrals work? | Females (mean, SD) | 4.16 (1.73) | Pre-clinical student (mean, SD) | 2.96 (1.65) | Medical student (mean, SD) | 3.83 (1.73) | White British (mean, SD) | 4.30 (1.71) |
|  | Males (mean, SD) | 4.12 (1.63) | Clinical student (mean, SD) | 4.62 (1.38) | Resident doctor (mean, SD) | 4.60 (1.50) | British Indian (mean, SD) | 3.92 (1.82) |
|  | P value | 0.3518 | P value | **<0.001*** | P value |  | P value | **<0.001*** |
| How confident are you in explaining to a patient how breast screening in the UK works? | Females (mean, SD) | 4.21 (1.63) | Pre-clinical student (mean, SD) | 3.30 (1.56) | Medical student (mean, SD) | 3.98 (1.61) | White British (mean, SD) | 4.31 (1.65) |
|  | Males (mean, SD) | 4.16 (1.54) | Clinical student (mean, SD) | 4.60 (1.36) | Resident doctor (mean, SD) | 4.47 (1.52) | British Indian (mean, SD) | 4.08 (1.68) |
|  | P value | 0.348 | P value | **p<0.001*** | P value | **<0.001*** | P value | **0.0212*** |
| How confident are you in identifying the red flags of breast cancer? | Females (mean, SD) | 4.64 (1.59) | Pre-clinical student (mean, SD) | 3.26 (1.53) | Medical student (mean, SD) | 4.18 (1.65) | White British (mean, SD) | 4.70 (1.64) |
|  | Males (mean, SD) | 4.57 (1.58) | Clinical student (mean, SD) | 4.95 (1.26) | Resident doctor (mean, SD) | 5.25 (1.24) | British Indian (mean, SD) | 4.47 (1.69) |
|  | P value | 0.3188 | P value | **<0.001*** | P value | **<0.001*** | P value | **<0.001*** |
| How confident are you in identifying patients who meet the 2 week wait referral for breast cancer? | Females (mean, SD) | 4.14 (1.74) | Pre-clinical student (mean, SD) | 2.76 (1.53) | Medical student (mean, SD) | 3.75 (1.75) | White British (mean, SD) | 4.18 (1.77) |
|  | Males (mean, SD) | 4.16 (1.66) | Clinical student (mean, SD) | 4.66 (1.38) | Resident doctor (mean, SD) | 4.73 (1.47) | British Indian (mean, SD) | 3.91 (1.81) |
|  | P value | 0.9706 | P value | **<0.001*** | P value | **<0.001*** | P value | **<0.001*** |
| How confident are you in counselling patients for family screening and counselling, after a diagnosis of breast cancer? | Females (mean, SD) | 3.49 (1.66) | Pre-clinical student (mean, SD) | 2.78 (1.54) | Medical student (mean, SD) | 3.39 (1.60) | White British (mean, SD) | 3.36 (1.64) |
|  | Males (mean, SD) | 3.61 (1.55) | Clinical student (mean, SD) | 3.95 (1.43) | Resident doctor (mean, SD) | 3.79 (1.61) | British Indian (mean, SD) | 3.62 (1.73) |
|  | P value | 0.1086 | P value | **<0.001*** | P value | **<0.001*** | P value | **0.035*** |
| How confident are you in breast anatomy, vasculature and lymphatic drainage? | Females (mean, SD) | 3.85 (1.61) | Pre-clinical student (mean, SD) | 3.96 (1.48) | Medical student (mean, SD) | 3.98 (1.54) | White British (mean, SD) | 3.87 (1.37) |
|  | Males (mean, SD) | 4.09 (1.53) | Clinical student (mean, SD) | 4.08 (1.52) | Resident doctor (mean, SD) | 3.94 (1.65) | British Indian (mean, SD) | 3.96 (1.61) |
|  | P value | **0.0039*** | P value | **<0.001*** | P value | 0.606 | P value | **0.0019*** |
| Rate your comfort level in addressing patients' emotional responses during consultations about breast conditions. | Females (mean, SD) | 4.33 (1.59) | Pre-clinical student (mean, SD) | 3.51 (1.53) | Medical student (mean, SD) | 3.87 (1.55) | White British (mean, SD) | 4.41 (1.59) |
|  | Males (mean, SD) | 4.10 (1.51) | Clinical student (mean, SD) | 4.21 (1.50) | Resident doctor (mean, SD) | 4.78 (1.40) | British Indian (mean, SD) | 4.26 (1.57) |
|  | P value | **0.0014*** | P value | **<0.001*** | P value | **<0.001*** | P value | **0.0292*** |

**Appendix 5: List of NHS trusts websites and resources analysed**

| England | Wales | Scotland | Northern Ireland |
| --- | --- | --- | --- |
| Airedale NHS Foundation Trust | Aneurin Bevan University Health Board | Ayrshire & Arran | Belfast Health and Social Care Trus |
| Alder Hey Children’s NHS Foundation Trust | Betsi Cadwaladr University Health Board | NHS borders | Northern Health and Social Care Trust |
| Ashford and St Peter’s Hospitals NHS Foundation Trust | Cardiff and Vale University Health Board | Dumfries & Galloway | Northern Ireland Ambulance Service HSC Trust |
| Avon and Wiltshire Mental Health Partnership NHS Trust | Cwm Taf Morgannwg University Health Board | Fife | South Eastern Health and Social Care Trust |
| Barking, Havering and Redbridge University Hospitals NHS Trust | Hywel Dda University Health Board | Forth Valley | Southern Health and Social Care Trust |
| Barnet Enfield and Haringey Mental Health NHS Trust | Powys Teaching Health Board | Grampian | Western Health and Social Care Trust |
| Barnsley Hospital NHS Foundation Trust | Swansea Bay University Health Board | Greater Glasgow & Clyde |  |
| Barts Health NHS Trust | Velindre University NHS Trust | Highland |  |
| Bedfordshire Hospitals NHS Foundation Trust | Welsh Ambulances Services NHS Trust | Lanarkshire |  |
| Berkshire Healthcare NHS Foundation Trust |  | [Lothian](https://www.scot.nhs.uk/organisations/lothian/) |  |
| Birmingham and Solihull Mental Health NHS Foundation Trust |  | Orkney |  |
| Birmingham Community Healthcare NHS Foundation Trust |  | Shetland |  |
| Birmingham Women’s and Children’s NHS Foundation Trust |  | Tayside |  |
| Black Country Healthcare NHS Foundation Trust |  | Western Isles |  |
| Blackpool Teaching Hospitals NHS Foundation Trust |  |  |  |
| Bolton NHS Foundation Trust |  |  |  |
| Bradford District Care NHS Foundation Trust |  |  |  |
| Bradford teaching hospital |  |  |  |
| Bridgewater Community Healthcare NHS Foundation Trust |  |  |  |
| Buckinghamshire Healthcare NHS Trust |  |  |  |
| calderdale and huddersfield nhs foundation trust |  |  |  |
| Cambridge University Hospitals NHS Foundation Trust |  |  |  |
| Cambridgeshire and Peterborough NHS Foundation Trust |  |  |  |
| Cambridgeshire Community Services NHS Trust |  |  |  |
| Central and North West London NHS Foundation Trust |  |  |  |
| Central London Community Healthcare NHS Trust |  |  |  |
| Chelsea and Westminster Hospital NHS Foundation Trust |  |  |  |
| Cheshire and Wirral Partnership NHS Foundation Trust |  |  |  |
| Chesterfield Royal Hospital NHS Foundation Trust |  |  |  |
| Cornwall Partnership NHS Foundation Trust |  |  |  |
| Countess of Chester Hospital NHS Foundation Trust |  |  |  |
| County Durham and Darlington NHS Foundation Trust |  |  |  |
| Coventry and Warwickshire Partnership NHS Trust |  |  |  |
| Croydon Health Services NHS Trust |  |  |  |
| Cumbria, Northumberland, Tyne and Wear NHS Foundation Trust |  |  |  |
| Dartford and Gravesham NHS Trust |  |  |  |
| Derbyshire Community Health Services NHS Foundation Trust |  |  |  |
| Derbyshire Healthcare NHS Foundation Trust |  |  |  |
| Devon Partnership NHS Trust |  |  |  |
| Doncaster and Bassetlaw Teaching Hospitals NHS Foundation Trust |  |  |  |
| Dorset County Hospital NHS Foundation Trust |  |  |  |
| Dorset Healthcare University NHS Foundation Trust |  |  |  |
| Dudley Integrated Health and Care NHS Trust |  |  |  |
| East and North Hertfordshire Teaching NHS Trust |  |  |  |
| East Kent Hospitals University NHS Foundation Trust |  |  |  |
| East Lancashire Hospitals NHS Trust |  |  |  |
| East Midlands Ambulance Service NHS Trust |  |  |  |
| East of England Ambulance Service NHS Trust |  |  |  |
| East Suffolk and North Essex NHS Foundation Trust |  |  |  |
| East Sussex Healthcare NHS Trust |  |  |  |
| Epsom and St Helier University Hospitals NHS Trust |  |  |  |
| Essex Partnership University NHS Foundation Trust |  |  |  |
| Frimley Health NHS Foundation Trust |  |  |  |
| Gateshead Health NHS Foundation Trust |  |  |  |
| George Eliot Hospital NHS Trust |  |  |  |
| Gloucestershire Hospitals NHS Foundation Trust |  |  |  |
| Gloucestershire Health and Care NHS Foundation Trust |  |  |  |
| Great Ormond Street Hospital For Children NHS Foundation Trust |  |  |  |
| Great Western Hospitals NHS Foundation Trust |  |  |  |
| Greater Manchester Mental Health NHS Foundation Trust |  |  |  |
| Guy’s and St Thomas’ NHS Foundation Trust |  |  |  |
| Hampshire Hospitals NHS Foundation Trust |  |  |  |
| Hampshire and Isle of Wight Healthcare NHS Foundation Trust |  |  |  |
| Harrogate and District NHS Foundation Trust |  |  |  |
| Herefordshire and Worcestershire Health and Care NHS Trust |  |  |  |
| Hertfordshire Community NHS Trust |  |  |  |
| Homerton Healthcare NHS Foundation Trust |  |  |  |
| Hounslow and Richmond Community Healthcare NHS Trust |  |  |  |
| Hull University Teaching Hospitals NHS Trust |  |  |  |
| Humber Teaching NHS Foundation Trust |  |  |  |
| Imperial College Healthcare NHS Trust |  |  |  |
| Isle of Wight NHS Trust |  |  |  |
| James Paget University Hospitals NHS Foundation Trust |  |  |  |
| Kent and Medway NHS and Social Care Partnership Trust |  |  |  |
| Kent Community Health NHS Foundation Trust |  |  |  |
| Kettering General Hospital NHS Foundation Trust |  |  |  |
| King’s College Hospital NHS Foundation Trust |  |  |  |
| Kingston and Richmond NHS Foundation Trust |  |  |  |
| Lancashire and South Cumbria NHS Foundation Trust |  |  |  |
| Lancashire Teaching Hospitals NHS Foundation Trust |  |  |  |
| Leeds Teaching Hospitals NHS Trust |  |  |  |
| Leeds Community Healthcare NHS Trust |  |  |  |
| Leeds and York Partnership NHS Foundation Trust |  |  |  |
| Leicestershire Partnership NHS Trust |  |  |  |
| Lewisham and Greenwich NHS Trust |  |  |  |
| Lincolnshire Community Health Services NHS Trust |  |  |  |
| Lincolnshire Partnership NHS Foundation Trust |  |  |  |
| Liverpool Heart and Chest Hospital NHS Foundation Trust |  |  |  |
| Liverpool University Hospitals NHS Foundation Trust |  |  |  |
| Liverpool Women’s NHS Foundation Trust |  |  |  |
| London Ambulance Service NHS Trust |  |  |  |
| London North West University Healthcare NHS Trust |  |  |  |
| Maidstone and Tunbridge Wells NHS Trust |  |  |  |
| Manchester University NHS Foundation Trust |  |  |  |
| Medway NHS Foundation Trust |  |  |  |
| Mersey Care NHS Foundation Trust |  |  |  |
| Mersey and West Lancashire Teaching Hospitals NHS Trust |  |  |  |
| Mid and South Essex NHS Foundation Trust |  |  |  |
| Mid Cheshire Hospitals NHS Foundation Trust |  |  |  |
| Mid Yorkshire Hospitals NHS Trust |  |  |  |
| Midlands Partnership University NHS Foundation Trust |  |  |  |
| Milton Keynes University Hospital NHS Foundation Trust |  |  |  |
| Moorfields Eye Hospital NHS Foundation Trust |  |  |  |
| Norfolk Community Health and Care NHS Trust |  |  |  |
| Norfolk and Norwich University Hospitals NHS Foundation Trust |  |  |  |
| Norfolk and Suffolk NHS Foundation Trust |  |  |  |
| North Bristol NHS Trust |  |  |  |
| North Cumbria Integrated Care NHS Foundation Trust |  |  |  |
| North East Ambulance Service NHS Foundation Trust |  |  |  |
| North East London NHS Foundation Trust |  |  |  |
| North London NHS Foundation Trust |  |  |  |
| Northern Care Alliance NHS Foundation Trust |  |  |  |
| North Middlesex University Hospital NHS Trust |  |  |  |
| North Staffordshire Combined Healthcare NHS Trust |  |  |  |
| North Tees and Hartlepool NHS Foundation Trust |  |  |  |
| North West Ambulance Service NHS Trust |  |  |  |
| North West Anglia NHS Foundation Trust |  |  |  |
| North West Boroughs Healthcare NHS Foundation Trust |  |  |  |
| Northampton General Hospital NHS Trust |  |  |  |
| Northamptonshire Healthcare NHS Foundation Trust |  |  |  |
| Northern Lincolnshire and Goole NHS Foundation Trust |  |  |  |
| Northumbria Healthcare NHS Foundation Trust |  |  |  |
| Nottingham University Hospitals NHS Trust |  |  |  |
| Nottinghamshire Healthcare NHS Foundation Trust |  |  |  |
| Oxford Health NHS Foundation Trust |  |  |  |
| Oxford University Hospitals NHS Foundation Trust |  |  |  |
| Oxleas NHS Foundation Trust |  |  |  |
| Pennine Care NHS Foundation Trust |  |  |  |
| Portsmouth Hospitals University NHS Trust |  |  |  |
| Queen Victoria Hospital NHS Foundation Trust |  |  |  |
| Rotherham Doncaster and South Humber NHS Foundation Trust |  |  |  |
| Royal Berkshire NHS Foundation Trust |  |  |  |
| Royal Brompton and Harefield NHS Foundation Trust |  |  |  |
| Royal Cornwall Hospitals NHS Trust |  |  |  |
| Royal Devon University Healthcare NHS Foundation Trust |  |  |  |
| Royal Free London NHS Foundation Trust |  |  |  |
| Royal National Orthopaedic Hospital NHS Trust |  |  |  |
| Royal Papworth Hospital NHS Foundation Trust |  |  |  |
| Royal Surrey NHS Foundation Trust |  |  |  |
| Royal United Hospitals Bath NHS Foundation Trust |  |  |  |
| Salisbury NHS Foundation Trust |  |  |  |
| Sandwell and West Birmingham Hospitals NHS Trust |  |  |  |
| Sheffield Children’s NHS Foundation Trust |  |  |  |
| Sheffield Health and Social Care NHS Foundation Trust |  |  |  |
| Sheffield Teaching Hospitals NHS Foundation Trust |  |  |  |
| Sherwood Forest Hospitals NHS Foundation Trust |  |  |  |
| Shrewsbury and Telford Hospital NHS Trust |  |  |  |
| Shropshire Community Health NHS Trust |  |  |  |
| Solent NHS Trust |  |  |  |
| Somerset NHS Foundation Trust |  |  |  |
| South Central Ambulance Service NHS Foundation Trust |  |  |  |
| South East Coast Ambulance Service NHS Foundation Trust |  |  |  |
| South London and Maudsley NHS Foundation Trust |  |  |  |
| South Tees Hospitals NHS Foundation Trust |  |  |  |
| South Tyneside and Sunderland NHS Foundation Trust |  |  |  |
| South Warwickshire NHS Foundation Trust |  |  |  |
| South West London and St George’s Mental Health NHS Trust |  |  |  |
| South West Yorkshire Partnership NHS Foundation Trust |  |  |  |
| South Western Ambulance Service NHS Foundation Trust |  |  |  |
| Southport and Ormskirk Hospital NHS Trust |  |  |  |
| St George’s University Hospitals NHS Foundation Trust |  |  |  |
| St Helens and Knowsley Teaching Hospitals NHS Trust |  |  |  |
| Stockport NHS Foundation Trust |  |  |  |
| Surrey and Sussex Healthcare NHS Trust |  |  |  |
| Surrey and Borders Partnership NHS Foundation Trust |  |  |  |
| Sussex Community NHS Foundation Trust |  |  |  |
| Sussex Partnership NHS Foundation Trust |  |  |  |
| Tameside and Glossop Integrated Care NHS Foundation Trust |  |  |  |
| Tavistock and Portman NHS Foundation Trust |  |  |  |
| Tees, Esk and Wear Valleys NHS Foundation Trust |  |  |  |
| Torbay and South Devon NHS Foundation Trust |  |  |  |
| The Christie NHS Foundation Trust |  |  |  |
| The Clatterbridge Cancer Centre NHS Foundation Trust |  |  |  |
| The Dudley Group NHS Foundation Trust |  |  |  |
| The Hillingdon Hospitals NHS Foundation Trust |  |  |  |
| The Newcastle Upon Tyne Hospitals NHS Foundation Trust |  |  |  |
| The Princess Alexandra Hospital NHS Trust |  |  |  |
| The Queen Elizabeth Hospital, King’s Lynn, NHS Foundation Trust |  |  |  |
| The Robert Jones and Agnes Hunt Orthopaedic Hospital NHS Foundation Trust |  |  |  |
| The Royal Orthopaedic Hospital NHS Foundation Trust |  |  |  |
| The Royal Marsden NHS Foundation Trust |  |  |  |
| The Royal Wolverhampton NHS Trust |  |  |  |
| The Walton Centre NHS Foundation Trust |  |  |  |
| University College London Hospitals NHS Foundation Trust |  |  |  |
| United Lincolnshire Hospitals NHS Trust |  |  |  |
| University Hospital Southampton NHS Foundation Trust |  |  |  |
| University Hospitals Birmingham NHS Foundation Trust |  |  |  |
| University Hospitals Bristol and Weston NHS Foundation Trust |  |  |  |
| University Hospitals Coventry and Warwickshire NHS Trust |  |  |  |
| University Hospitals of Derby and Burton NHS Foundation Trust |  |  |  |
| University Hospitals Dorset NHS Foundation Trust |  |  |  |
| University Hospitals of Leicester NHS Trust |  |  |  |
| University Hospitals of Morecambe Bay NHS Foundation Trust |  |  |  |
| University Hospitals of North Midlands NHS Trust |  |  |  |
| University Hospitals Plymouth NHS Trust |  |  |  |
| University Hospitals Sussex NHS Foundation Trust |  |  |  |
| Walsall Healthcare NHS Trust |  |  |  |
| Warrington and Halton Teaching Hospitals NHS Foundation Trust |  |  |  |
| West Hertfordshire Hospitals NHS Trust |  |  |  |
| West London NHS Trust |  |  |  |
| West Midlands Ambulance Service NHS Foundation Trust |  |  |  |
| West Suffolk NHS Foundation Trust |  |  |  |
| Whittington Health NHS Trust |  |  |  |
| Wirral Community NHS Foundation Trust |  |  |  |
| Wirral University Teaching Hospital NHS Foundation Trust |  |  |  |
| Worcestershire Acute Hospitals NHS Trust |  |  |  |
| Wrightington, Wigan and Leigh NHS Foundation Trust |  |  |  |
| Wye Valley NHS Trust |  |  |  |
| York and Scarborough Teaching Hospitals NHS Foundation Trust |  |  |  |
| Yorkshire Ambulance Service NHS Trust |  |  |  |
